# Linking clinical trial data with real-world data for enhanced clinical evidence generation: methodological considerations and recommendations

**DOI:** 10.3389/fphar.2026.1887249

**Published:** 2026-08-07

**Authors:** Anna-Katharina Meinecke, John Diaz-Decaro, Kathleen M. Gavin, Tianyu Sun, Jordan B. Strom, Montse Soriano Gabarró, Hu Li, Dana Y. Teltsch, Mehdi Najafzadeh, Catherine A. Panozzo, Pareen Vora, Mehmet Burcu

**Affiliations:** 1 Bayer AG, Berlin, Germany; 2 ModernaTX, Inc., Cambridge, MA, United States; 3 Datavant, Inc., Phoenix, AZ, United States; 4 Division of Cardiovascular Medicine, Beth Israel Deaconess Medical Center, Harvard Medical School, Boston, MA, United States; 5 Independent Researcher, Potsdam, Germany; 6 Neurocrine Biosciences, San Diego, CA, United States; 7 EviDT LLC, Lexington, MA, United States; 8 UCB, Inc, Cambridge, MA, United States; 9 Merck & Co., Inc., Rahway, NJ, United States

**Keywords:** clinical trial data, data linkage, privacy preserving record linkage, real world data, RWD, tokenization

## Abstract

Randomized controlled trials (RCTs) remain the cornerstone of causal inference on the safety and efficacy of medicinal products. But their limited follow-up, controlled settings, and narrowly defined data collection to balance the burden to patients and maintain study feasibility often results in unaddressed important questions for health authorities and other clinical decision makers. Linkage of RCT data to routinely collected health data [real-world data (RWD)] offers a mechanism for addressing these gaps by extending observations and outcomes assessment into routine practice. This paper synthesizes methodological and operational considerations for RCTs with RWD linkage, drawing on deterministic, probabilistic, referential, and privacy-preserving record linkage methodologies and on four case studies that span different indications and regional settings: a long-term follow-up of a human papillomavirus vaccine trial using deterministic linkage via universal Personal Identity Numbers; a U.S. linkage of a respiratory syncytial virus vaccine trial to administrative health claims using privacy-preserving tokenization; and two transcatheter aortic valve replacement trials linked to Medicare claims to reproduce randomized treatment-effect estimates and to assess transportability to the broader Medicare population. We offer actionable methodological considerations and recommendations, drawing on the case studies and the broader linkage literature. Progress beyond the current state will require global health authority guidance specific to linking clinical trials with routinely collected data, shared benchmarks for evaluating linkage approaches, routine reporting and assessments of impact of use cases on regulatory and reimbursement decisions. Linkage of clinical trial data with routinely collected data sources can meaningfully accelerate clinical development and inform health authority and other clinical decision making.

## Introduction

Regulatory, health technology assessment (HTA), reimbursement, clinical and patient decisions increasingly require evidence that extends beyond the population, treatment duration and outcomes assessed in randomized clinical trials. While RCTs remain the cornerstone of causal inference, their limited follow-up, controlled settings, and narrowly defined data collection often leaves important unaddressed questions across the different stages of clinical development. Bridging RCT evidence with routinely collected health data [real-world data (RWD)] offers an opportunity to address these gaps by extending observation and assessment of treatment outcomes into routine clinical practice. In an RCT, randomization of patients into treatment and control groups and a wide array of data including demographics, medical history, laboratory results, treatment exposure, and patient-reported outcomes are prospectively collected to investigate safety and efficacy of an intervention. While this structured data collection supports internal validity, it is also resource-intensive, operationally complex, and necessarily constrained to a predefined set of variables captured within trial-specific electronic data capture (EDC) systems (Califf et al., 2016; Concato and Corrigan-Curay, 2022). Real-world evidence (RWE) studies, in contrast, usually use secondary healthcare data collected from sources such as electronic health records, administrative claims databases, vital statistics, and disease registries to evaluate wide range of scientific and clinical questions and treatment outcomes. These include, but are not limited to, safety and effectiveness assessments, healthcare resource utilization, cost effectiveness, risk prediction and surveillance across diverse treatments including drugs, vaccines, and medical devices (Dang, 2023).

Integrating individual-level clinical trial data with RWD sources can provide several important benefits for enhanced evidence generation, including extended follow-up, access to outcomes and healthcare utilization data not routinely captured within clinical trial electronic data capture systems, and reduced reliance on additional site-based data collection (Eckrote et al., 2024). By leveraging routinely collected healthcare data, such integration can support more efficient follow-up, enable enrichment analyses, and enhance the assessment of longer-term and additional outcomes that are not usually collected in traditional RCT settings. Linking trial data with RWD can also accelerate RWE generation by allowing real-world outcomes to be analyzed concurrently with, or soon after, trial readout, rather than waiting for trial completion to launch a separate stand-alone RWE study (Rogers et al., 2021).

Unlike RCT extension studies which prolong follow-up within a clinical trial framework and require additional burden on participants and study sites, trial linkage with RWD leverage existing healthcare data sources to prospectively track the treatment outcomes over extended periods of time without requiring active participant follow-up (NajafZadeh et al., 2025; Burcu et al., 2022). Such linkage can complement RCT-collected medical history and capture healthcare encounters or treatment outcomes in real-world settings, that occur before (Fillit et al., 2024) or after the trial but are not routinely captured within the RCT setting (Fillit et al., 2024).

As the regulatory, HTA, reimbursement and clinical practice increasingly emphasize the need for additional outcomes, durability of treatment effects and long-term outcomes (Hennessy et al., 2025), there is growing multi-stakeholder interest in enhanced evidence generation through linkage of trial data with RWD. However, the feasibility and validity of RCT–RWD linkage studies hinge on successfully addressing operational and methodological challenges that vary across geographies, data infrastructures, and use cases.

The ability of RCT–RWD linkage studies to inform healthcare decision-making depends on alignment across four interdependent domains:

- The decision context in which evidence will be used;
- The linkage performance (measured by relevant metrics) and properties;
- Governance and privacy-preserving implementation; and
- Operational feasibility, and availability of reliable high quality RWD sources relevant to the underlying clinical and regulatory context.

This paper provides a methodological commentary with illustrative case studies of RCT-RWD data linkage options, and proposes actionable recommendations to maximize the use and value of data linkages.

### Data linkage methodologies: definitions and approaches

An increasing number of RCTs are incorporating linkage to RWD. Depending on the clinical, regional/national, and regulatory context, various methods for RCT data and RWD linkages can be selected. Here, we focus on methodologies for linking RCT data to routinely collected healthcare data that have been aggregated into structured datasets, rather than on the retrieval and abstraction of patient-level (or mediated) medical charts, although this important method for integrating RWD into RCTs is not detailed here. Deterministic and probabilistic, the two most common types of linkage, as well as referential matching are approaches that are briefly explained below. Furthermore, privacy preserving record linkage (PPRL), a methodology underlying data linkage without needing to share a patient’s direct identifiers, is introduced.

#### Deterministic linkage

Deterministic linkage involves matching datasets using exact patient-level identifiers, such as date of birth or national health identifier (ID). It can be performed through single-step strategies, where all identifiers are compared at once, or multi-step strategies, which allow for matching criteria using agreement on a set of predefined identifiers. While this method is precise and efficient for large datasets, it relies on the availability of national or regional identifiers and may face challenges related to data access and legal restrictions (Dusetzina et al., 2014).

#### Probabilistic linkage

Probabilistic linkage employs statistical methods to estimate the likelihood of a match between records based on multiple non-unique/ partially identifying attributes, such as name and address. It assigns weights to these attributes and calculates probability scores to classify records as matches or non-matches. This approach is particularly beneficial when unique identifiers are missing or unreliable, allowing for flexible integration of disparate data sources, though it is more computationally intensive and may require manual validation of uncertain matches (Bohensky et al., 2010; Doidge and Harron, 2018; Harron et al., 2017; Pratt et al., 2020; Sayers et al., 2016).

#### Referential matching

Referential matching can enhance match accuracy by using an external reference database, such as large credit reporting agencies, to supplement incomplete or inconsistent demographic data providing a multi-record benchmark for matching (Grannis et al., 2022). This method provides a comprehensive view of an individual’s identity, helping to resolve ambiguities in record linkage. Referential data can be incorporated into deterministic, probabilistic, or hybrid matching workflows to improve match performance.

#### Privacy-preserving record linkage

PPRL is a method that allows two or more parties to link datasets from the same individual across different data sources accurately without revealing sensitive personally identifiable information (PII) (Bernstam et al., 2022; Tyagi and Willis, 2025). Whereas the above outlined linkage methods define how matches are decided using direct or indirect identifiers, PPRL methods modify how identifiers are presented before linkage takes place. PPRL uses transformations such as data encryption, hashing, bloom filters or tokenization to ensure datasets are matched across data sources without revealing PII. Although PPRL enhances data protection and facilitates secure data sharing, it requires applying encryption techniques, adding a layer of complexity. Furthermore, because each PPRL implementation applies its own approach to transforming identifiers, the resulting tokens are rarely interoperable across systems. Increasingly, health systems, manufacturers, and data partners are participating in commercial PPRL/tokenization ecosystems. Standardizing on a common token framework across multiple studies can increase efficiency, consistency and harmonization, and reduce cost, and re-linkage/validation burden.

#### Choosing the appropriate linkage method

The choice of a data linkage method for a given RCT-RWD study is usually made on a case-by-case basis, taking into account feasibility factors such as quality of the linkage data (e.g., missingness or errors), the uniqueness of the available identifiers, the relative consequences of false positive links vs. false negative links, the availability of linkage software program, computational resources, and local privacy regulations (Harron, 2022; Ong et al., 2024). In addition, linkage method selection may also account for whether the effort is study-specific or programmatic in scope. For one-off projects, decisions may prioritize immediate performance and feasibility; however, for portfolios of studies, sustainability, repeatability, and cross-domain reuse become more important. Establishing an interoperable token-based system can enable repeated linkage without reprocessing raw identifiers, support use across therapeutic areas, accelerate activation of new studies, and promote consistent governance and auditability within secure research environments as described below. See Table 1 for more information regarding selecting linkage methodologies.

**TABLE 1:** Choosing a linkage method.

Method	Mechanism	When to use	Key references
Deterministic linkage	Exact match on unique identifiers	Best when high-quality, stable unique identifiers (or near-unique combinations) are available and missed matches are more tolerable than false matches. Hybrid deterministic-probabilistic approaches often start here for high-confidence pairs	Harron (2022), Ong et al. (2020), Zhu et al. (2015)
Probabilistic linkage	Weighted scoring across partial identifiers	Preferred when identifiers are error-prone, incomplete, or inconsistently formatted, and explicit control over the false-match vs. missed-match trade-off (via thresholding) with evaluation is needed	Harron (2022)
Referential linkage	Reference-augmented matching	Most appropriate when a high-quality, comprehensive reference dataset is available (e.g., large demographic reference files or enterprise master patient indexes), particularly when linkage occurs long after identifier collection and demographic attributes may have changed (e.g., address, name). Can improve sensitivity while maintaining very high PPV, though performance depends on the coverage, accuracy, and recency of the reference database	Grannis et al. (2022)
Privacy preserving record linkage	Tokenized/Encrypted-PII	Most appropriate when direct identifiers cannot be shared across organizations due to privacy regulations or contractual constraints, such as U.S. trial-to-claims linkages and multi-site research networks requiring HIPAA-compliant linkage without exposure of personal identifiers	Bernstam et al. (2022), Tyagi and Willis (2025)

Abbreviations: GDPR, general data protection regulation; HIPAA, health insurance portability and accountability act; PII, personally identifiable information; PPV, positive predictive value; PPRL, privacy-preserving record linkage.

Ultimately, the most commonly published method for record linkage in clinical trials has been deterministic linkage (NajafZadeh et al., 2025), but probabilistic methods are also used (Hay et al., 2019). Despite increasing adoption, the use of PPRL technologies in RCT-RWD linkage projects remains underrepresented in the published literature. Although PPRL and tokenization have gained traction since 2020 as mechanisms to enable linkage across fragmented health systems without exchanging personally identifiable information (Kiernan et al., 2022; Marsolo et al., 2023; Pathak et al., 2024), their limited visibility in published RCT linkage projects likely reflects the lag between methodological uptake, trial conduct, and publication, with further reports expected in the coming years (NajafZadeh et al., 2025).

Finally, RCTs that link RWD should also aim to complete the Consolidated Standards of Reporting Trials (CONSORT) extension for randomized controlled trials conducted using cohorts and routinely collected data (CONSORT-ROUTINE) (Kwakkenbos et al., 2021). According to these standards, authors should describe if the study included data linkage across two or more databases and, if so, detail the linkage techniques and methods used to evaluate completeness and accuracy of the linkage.

### Operational considerations for RCT-RWD linkages

Linking RWD with data from RCTs is often operationally complex and subject to constraints that extend beyond methodological considerations alone. Many of the factors that shape the feasibility, validity, and interpretability of linkage studies, such as data availability and access, governance frameworks, and infrastructure, are partially or entirely outside the direct control of investigators. Failure to account for these operational considerations can limit the ability of RCT–RWD linkage studies to generate robust and timely evidence using appropriate linkage methodologies.

Several key areas of operational considerations should be addressed when designing and implementing RCT–RWD linkage studies including:

- Data availability, relevance, reliability, and traceability
- Legal, regulatory, compliance and bioethical considerations including stakeholder engagement
- Infrastructure and technological requirements for large-scale data linkage
- Operational readiness, including trial protocol, informed consent, site onboarding, and training

#### Navigating complexity around RWD availability, data fitness and traceability

The availability, relevance, reliability, and traceability of RWD are highly dependent on the source data and linkage methods employed, making these foundational considerations in RCT–RWD linkage studies. It is essential to ensure that key study outcomes are not only available within a given RWD source but are also captured with sufficient quality. Moreover, data are considered fit-for-purpose when they are relevant and reliable to address a specific research or regulatory question, with sufficient completeness, accuracy, and transparency to support valid and auditable inference (FDA, 2023). Because RWD are collected as part of routine care and not for research purposes, data quality challenges such as missingness, duplication and inconsistent records are common. Typographical errors, incomplete fields, and changes in identifiers over time may reduce the reliability of a dataset for linkage (Bohensky et al., 2010).

Importantly, missingness is not limited to individual data elements; entire patient subgroups may be underrepresented or absent from certain RWD sources. For example, heterogeneity in the U.S. insurance landscape or fragmentation across care settings can result in systematic gaps in coverage for specific populations (Sandhu et al., 2021). Furthermore, it is important to recognize the potential for bias introduction when linkage success varies across patient groups, healthcare providers, or data sources, particularly when linkage failure is correlated with sociodemographic or clinical characteristics (Harron et al., 2017). For example, in U.S.-based settings, individuals lacking continuous medical insurance coverage or receiving care primarily in community-based clinics may be underrepresented or entirely absent from certain claims or electronic health record (EHR) datasets, resulting in systematic differences between linked and non-linked populations and potentially biased estimates of treatment effects or outcomes. These obstacles are further amplified in multi-country or cross-system linkage efforts, where differences in coding practices and data standards must be addressed (Bhatt, 2023; Eckrote et al., 2024; Liaw et al., 2021; McKinsey, 2023; Rogers et al., 2021).

Investigators should assess data provenance and traceability, including how data are generated, curated, transformed, and normalized before linkage, as these factors affect interpretability and analytic validity. Detailed metadata on transformation, normalization, and harmonization are critical for transparency and reproducibility, although access to source data is often constrained by permissions and governance frameworks (McKinsey, 2023; Rudrapatna and Butte, 2020).

#### Legal, regulatory compliance and bioethical considerations and stakeholder engagement

The linkage of patient-level RCT and RWD requires careful consideration of legal, regulatory, and bioethical frameworks that govern the collection, use, and secondary use of health data. Data governance requirements vary substantially across jurisdictions, with important differences between frameworks such as the General Data Protection Regulation (GDPR) in the European Union, the Health Insurance Portability and Accountability Act (HIPAA) in the United States, and country-specific regulations such as Japan’s Act on the Protection of Personal Information (Aidun, 2025; Personal Information Protection Commission, 2022). These differences influence not only the permissibility of data linkage, but also cross-border data transfers, localization requirements, and the operational models through which linkage can be conducted in multi-regional clinical trials.

Beyond privacy law, regulatory compliance must also address the interaction between trial-generated data and externally sourced RWD. In some implementations, linkage or tokenization activities may capture data elements that overlap temporally or conceptually with those collected as part of the pivotal clinical study, creating partially overlapping datasets that risk generating dual “sources of truth” for the same period of observation. Such overlap can introduce ambiguity in endpoint derivation, data reconciliation, audit trails, and regulatory submission documentation. In RCT–RWD linkage studies, clear delineation of data provenance, advance specification of which dataset constitutes the authoritative source for particular variables or time periods, and alignment with Good Clinical Practice (ICH E6 [R3]) and regulatory expectations for RWD reliability and transparency are essential to preserve data integrity, minimize ambiguity during periods of dataset overlap, and ensure inspection readiness (Berger et al., 2017; EMA, 2025; FDA, 2023; ICH, 2025; Kwakkenbos et al., 2021).

Bioethical considerations also play a central role in the design and implementation of RCT–RWD linked studies. Ethical evaluation should address respect for persons, protection of privacy, equity in data use, and transparency in governance and data stewardship practices. It is also essential to ensure that patient consent processes are appropriate and clearly articulated, including whether consent for data linkage is explicit or waived, how data will be used and shared, and what safeguards are in place to protect participants and their data. The ethical implications of linkage may also differ depending on whether linkage is embedded prospectively within a RCT or conducted retrospectively as secondary use of existing data, as these contexts shape participant expectations, oversight requirements, and acceptable safeguards. Operational elements such as consent models are discussed in the following section.

Developing and implementing a stakeholder engagement strategy early in the study lifecycle is an important consideration for ensuring that linkage approaches are aligned with regulatory and decision-making expectations. Expectations for data linkage may vary across jurisdictions and among regulators, HTA bodies, payers, and ethics committees. For multinational trials, linkage planning should begin early and explicitly account for heterogeneity in identifiers, consent requirements, governance, coding systems, and endpoint definitions across countries and sites. At present, RCT-RWD linkage in multinational studies remains challenging, and use cases are still limited. As illustrated in the use cases described in later sections of this paper, linkage was achieved only in selected jurisdictions and for a subset of patients, yet it nevertheless yielded substantial additional value for evidence generation. In many settings, a hybrid approach is more likely to be feasible, while allowing country- or site-specific implementation to reflect local legal and data access constraints. Looking ahead, broader cross-sector collaboration and continued infrastructure development will be needed to establish common standards and guiding principles for scalable implementation. Importantly, early stakeholder engagement can help identify jurisdiction-specific requirements, areas of alignment, and potential constraints (“no-go” areas) before linkage activities are initiated (Eckrote et al., 2024; Sagkriotis, 2026).

#### Infrastructure and technological requirements for large-scale data linkage

Appropriate infrastructure and technological capabilities are central to enabling large-scale RCT-RWD linkages particularly in settings where data governance, privacy protections, and cross-organizational collaboration may impose constraints on data movement and access. Record-level linkage requires secure identity resolution workflows, robust data management systems, proactive privacy management, and clearly defined governance controls.

Increasingly, linkage activities are conducted within secure, cloud-based environments that function as Trusted Research Environments (TREs), providing controlled access to sensitive data while supporting auditability, reproducibility, and compliance with applicable regulatory requirements. Such environments enable linkage to occur without unnecessary duplication or uncontrolled distribution of identifiable data and allow comprehensive logging of data access, transformation, and analytic workflows. Even though not currently used for industry sponsored RCT linkage, national-scale platforms illustrate how infrastructure and governance can be co-designed to support secure large-scale linkage. Examples include the All of Us Researcher Workbench in the United States (NIH, 2024), the UK Biobank Research Analysis Platform (UK Biobank, 2025), and Finland’s Findata (Findata, 2025), which centrally governs access to linked health and social data under strict data protection legislation.

At scale, linkage infrastructure must support secure tokenization or identity resolution processes, metadata capture to preserve data provenance, version control, and clearly defined authoritative data sources when multiple datasets overlap temporally or conceptually. Maintaining detailed lineage documentation and auditable workflows is essential to ensure transparency, reproducibility, and inspection readiness when linked datasets contribute to clinical development decisions or regulatory submissions.

Emerging applications of artificial intelligence (AI) and machine learning (ML) may potentially further support large-scale linkage efforts by improving data quality assessment, identifying anomalous records, and mitigating linkage-related risks. For example, ML methods can be used to detect outliers, inconsistencies, or systematic gaps in health data that may affect linkage success or downstream analyses (Janoudi et al., 2024). While such techniques hold promise, their application in RCT–RWD linkage contexts must be carefully evaluated to ensure credibility, trustworthiness, interpretability, and alignment with regulatory and ethical expectations. Infrastructure that prioritizes transparency, validation, and governance alongside technical capability and privacy preservation is therefore critical to the successful and responsible use of RCT–RWD integration. In addition, AI/ML can help extract meaningful information from unstructured data sources (e.g., clinical notes, reports), enhancing the completeness and utility of linked datasets.

#### Operational considerations for the implementation of linking RCT and RWD and site readiness: trial protocol, informed consent, onboarding and training

Ideally, linkage feasibility should be assessed early in the development process, before protocol finalization and certainly before operational implementation. Country laws, privacy regulations, consent requirements, technical feasibility, as well as data availability, quality, reliability and accurate linkage capability inherently require early evaluation so that relevant provisions can be incorporated into the core protocol, consent materials, data management plan, and statistical analysis plan (Batech et al., 2025). However, we acknowledge that the need for RCT-RWD linkage may emerge after trial initiation. When this occurs, sponsors and investigators should conduct a prompt feasibility assessment and, if linkage remains valuable, proceed with rapid planning to address governance, data harmonization, and analytical requirements.

Details on data linkage may need to be incorporated into the trial protocol, data management plan (DMP) and the statistical analysis plan (SAP) for transparency and to support subsequent evaluation by health authorities and other healthcare decision makers. Decisions regarding consent language, patient engagement, site readiness, and linkage infrastructure are interdependent and are best addressed prospectively during RCT design and RWE planning rather than retrofitted after trial completion. Early integration of RWD linkage into the protocol and informed consent can improve operational feasibility by enabling collection of required permissions and identifiers at enrollment, while minimizing disruption, participant burden, and risks to trial conduct or regulatory compliance. By contrast, retrospective linkage is less flexible; inconsistent or absent consent for linkage and secondary use can reduce eligibility and introduce selection bias.

Optimizing informed consent for RCT–RWD linkage requires clear communication of purpose, safeguards, risks, benefits and participant protections while minimizing technical jargon. Partnering with patients, patient advocacy groups and community stakeholders can improve consent design, clarify educational materials, and build trust. Key considerations also include whether to restrict data linkage to a specific duration or set of use cases, the choice between opt-in versus opt-out approaches and the scope of consent for secondary or future use of data (Arellano et al., 2023).

Site onboarding and training represent additional operational considerations. Study sites must be adequately prepared to inform participants about data linkage activities, obtain valid informed consent, and collect any required identifiers or linkage-related information at enrollment. Ongoing monitoring of consent rates and identifier completeness can help identify implementation gaps early and prevent downstream linkage failures.

Finally, implementation requires selection of a governance-compliant, record linkage approach (e.g., consistent with GDPR or HIPAA requirements) and identification of reliable and relevant RWD sources with sufficient overlap.

### Case examples of RCTs with linkages to RWD sources

The implementation of RCT-RWD linkages is highly context-specific and influenced by therapeutic or disease area, regional/national infrastructure, data availability, and regulatory requirements. We present four case studies spanning various indications and regional/national settings, including two regulatory-facing studies (one in European countries and the other in the U.S.) and two methodological assessment studies.

#### Case study 1: linkage of RCT data to prospectively collected registry and other RWD sources for long-term follow-up in Europe

The FUTURE II trial (Enerly et al., 2020) was a pivotal phase 3 RCT that demonstrated the 4-year efficacy, immunogenicity, and safety of a quadrivalent human papillomavirus (qHPV) vaccine in women between ages 15 and 26 years. Participants from >10 countries were enrolled in this trial. Given the anticipated long latency between HPV infection and high-grade cervical disease and HPV-related cancer incidence, longer term observation was necessitated to confirm long-term effectiveness, safety and immunogenicity of the vaccine.

**FIGURE 1:**
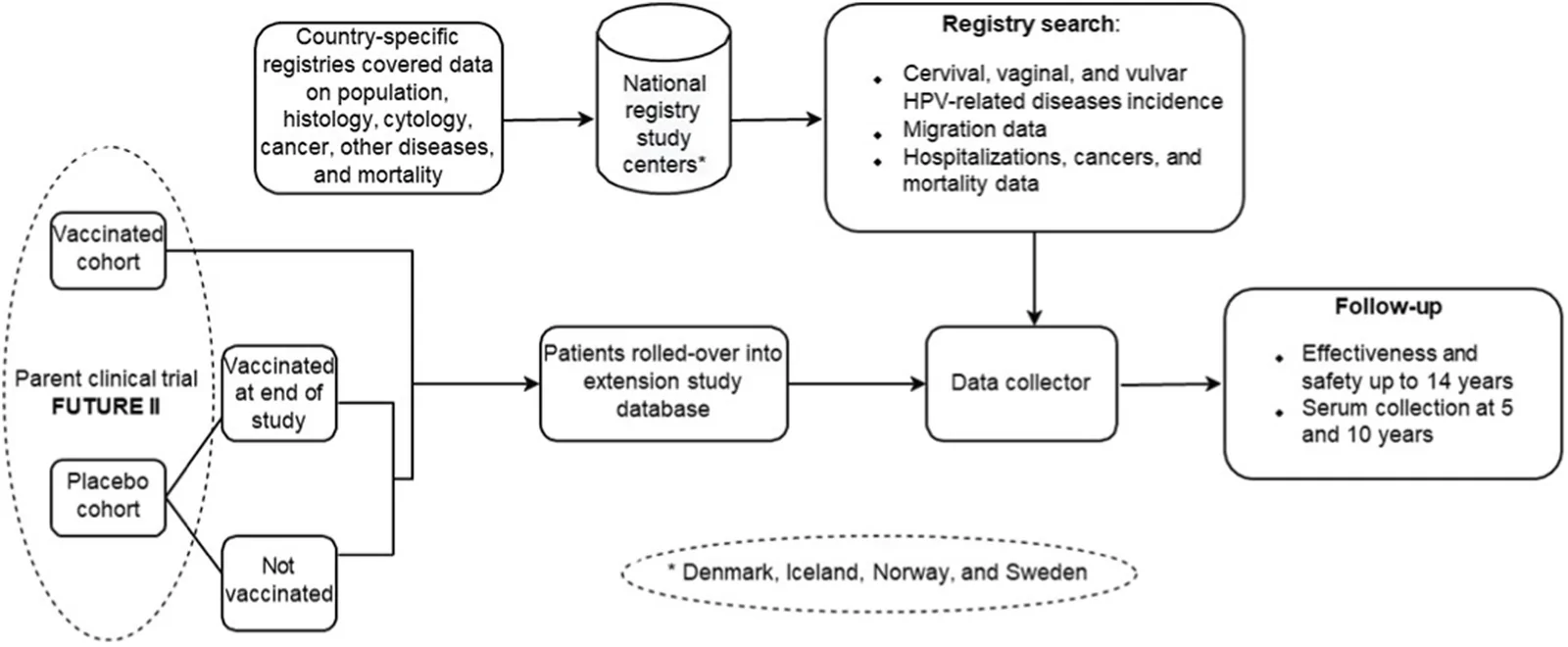
Linkage of trial patients to prospectively collected real-world data in Nordic countries in a clinical trial extension use case for long-term follow-up and outcomes* *Note: Figure adapted from Su et al. (2025), Enerly et al. (2020).

A post-marketing long-term follow-up (LTFU) study extended the FUTURE II phase 3 trial to 14 years in selected Nordic European countries, namely, Denmark, Iceland, Norway and Sweden, to evaluate vaccine effectiveness, against HPV-related cervical precancers and cancers, long-term immunogenicity and safety (Burcu et al., 2022; Enerly et al., 2020). As illustrated in Figure 1 (Su et al., 2025; Burcu et al., 2022; Enerly et al., 2020), the study utilized the national health registries, in which individual patient records were linked via deterministic linkage by a unique Personal Identity Number (PIN). By linking each participant’s unique PIN to national health registries, the LTFU achieved almost complete capture of clinical outcomes and laboratory specimens, showing that registry-based extension design can potentially satisfy post-marketing requirements and related evidence needs (Su et al., 2025). Depending on different national requirements, either the informed consent for LTFU was covered in the base parent trial consent or an additional informed consent was required.

The LTFU tracked the following three patient groups from the original parent trial (Enerly et al., 2020):

- Cohort 1, women who were originally randomized to receive vaccination during the parent trial
- Cohort 2, women randomized to receive placebo who then accepted and received catch-up qHPV vaccination at unblinding (at the end of the parent trial)
- Not-Vaccinated group, a small subgroup of women originally randomized to placebo at the parent trial who later declined vaccination at unblinding.

All groups were prospectively followed through registries while participants also attended clinic visits for serum collection and biopsy re-analysis. Country-specific registries were utilized to collect the necessary data for effectiveness, immunogenicity, and safety surveillance. The study organization included National Registry Study Centers (NRSCs) in each country to collect national data, and a Nordic Coordination Center (NCC) in Norway to coordinate specimen flow. Effectiveness and safety analyses were performed every 2 years, with serum collection follow-up conducted twice, approximately 5 and 10 years after the end of the parent study (Enerly et al., 2020). The long-term evidence generated through this registry-based linkage informed ongoing benefit–risk characterization of the qHPV vaccine and contributed to the post-marketing evidence base to support health authority and other clinical decision making.

#### Case study 2: linkage of RCT data to medical and pharmacy claims for accelerated capture of additional health and economic outcomes in a US setting

The ConquerRSV trial (NCT05127434, 2021) was a randomized, double-blind, placebo-controlled phase 2/3 pivotal trial evaluating a single-dose RSV vaccine in adults ≥ 60 in 22 countries (Wilson et al., 2023). As an exploratory objective of the ConquerRSV Trial, the ROSE study (NCT05572658, 2022) linked U.S. trial participants to medical claims using PPRL. This approach was designed to enable evaluation of outcomes not collected in the parent ConquerRSV trial, including respiratory, cardiovascular, and RSV-specific hospitalizations, healthcare costs and utilization, disease exacerbations among participants with pre-existing asthma, chronic obstructive pulmonary disease (COPD), or congestive heart failure (CHF).

ROSE operated under a separate protocol and consent process, allowing optional participation independent of the parent trial. Enrollment began approximately 10 months after ConquerRSV initiation and continued for 3 months beyond the parent trial’s accrual period. Of 136 U.S. ConquerRSV sites, 109 participated in ROSE (93% of responding sites expressed interest); across these sites, 10,994 individuals consented to linkage (67% of participants at participating sites). Of those consented, 4,242 (38.6%) were successfully linked to Medicare fee-for-service (FFS) claims data or selected commercial/Medicare Advantage claims. Uninsured participants were not captured.

**FIGURE 2:**
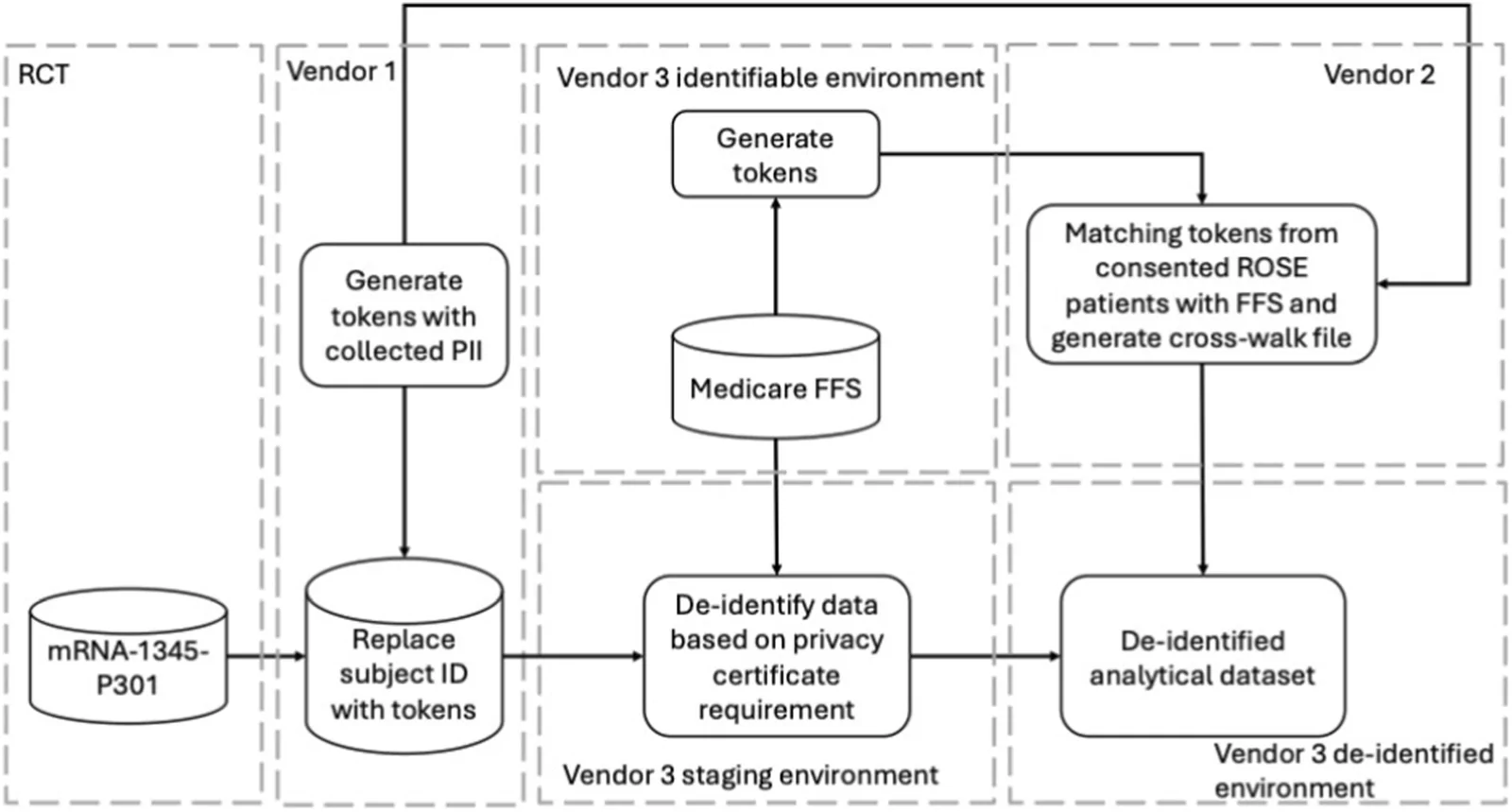
Overview of the ROSE (mRNA-1345-P901) Study Design: Privacy-preserving linkage of Real-World Data to ConquerRSV Trial Participants in the United States. Abbreviations: RCT, randomized controlled trial; PII, personally identifiable information; FFS, Fee-For-Service.

As illustrated in Figure 2, limited personally identifiable information (first and last name, date of birth, zip code, and sex) was used solely to generate encrypted tokens, with only tokens and study subject IDs transmitted for linkage. A formal data privacy certification was obtained to ensure that incorporation of select ConquerRSV variables would not compromise de-identification of the claims data sources or violate data-use agreements.

In the linked cohort, baseline demographic and clinical characteristics were generally well balanced between vaccine and placebo groups after linkage, as shown in Supplementary Table S1, with a modest imbalance observed only in diabetes prevalence (21.9% vs. 18.1%). Nonetheless, unique challenges were encountered in attempting to conduct ROSE. First, the fragmented U.S. insurance system limited successful linkage to only 38.6% of consented participants. Even with a low linkage rate, there was no evidence suggesting that randomization was broken as the vaccine and placebo groups remained well-balanced across measured baseline characteristics (Supplementary Table S1). Second, endpoint ascertainment differed between actively monitored clinical trial and passively captured claims. Thus, seemingly similar endpoints could have numeric discrepancies across sources and should be interpreted with caution. While tokenization-based linkage offers strengths, anticipating and addressing these practical challenges is critical for future RCT–RWD linkage studies in the U.S.

Beyond its scientific outputs, ROSE provided proof-of-concept evidence on the feasibility, performance, and limitations of PPRL-based RCT–RWD linkage in the fragmented U.S. payer environment, generating operational lessons that would inform prospective integration of linkage planning in subsequent trial designs.

#### Case study 3: linkage of RCT data from patients to medical claims to reproduce treatment effect in a US setting

The CoreValve Pivotal trials comprised several randomized comparisons of transcatheter aortic valve replacement (TAVR) using the self-expanding Medtronic CoreValve bioprosthesis versus surgical aortic valve replacement (SAVR) in severe symptomatic aortic stenosis, enrolling high-risk (HiR, NCT01240902, 2010), intermediate-risk (SURVATI, NCT01586910, 2012), and low-risk (NCT02701283, 2016) patients. Under the NHLBI-funded EXTEND initiative (1R01HL136708 (Strom et al., 2019), Medicare FFS inpatient claims from 2011 to 2016 were deterministically linked to trial participants (HiR and SURTAVI) using demographic and clinical information (age, date of birth, sex, admission and discharge dates, procedure date and type, and hospital name), excluding patients younger than 65 years and those treated at Veterans Administration or international sites (N = 479) (Strom et al., 2019). Linkage was successful for 80% of HiR (600/750) and 60.5% of SURTAVI (1005) participants, with nonmatches largely attributable to Medicare Advantage enrollment. Trial events were matched to claims within a ±14-day window, selecting the closest date when multiple candidates were present. In linked cohorts, primary outcomes reproduced the randomized effect estimates: in HiR, 1-year mortality was similar across data sources (trial HR 0.84, 95% CI 0.65–1.09, p = 0.33; claims HR 0.86, 95% CI 0.66–1.11, p = 0.34; interaction p = 0.80), confirming noninferiority of TAVR versus SAVR; in SURTAVI, the 2-year composite of death or stroke likewise aligned (trial HR 1.08, 95% CI 0.79–1.48, p = 0.90; claims HR 1.02, 95% CI 0.73–1.41, p = 0.58; interaction p = 0.89), again supporting noninferiority. For secondary outcomes in HiR, treatment effect estimates were similar for death, acute kidney injury, aortic valve reintervention, transient ischemic attack, and pacemaker placement but more discrepant for stroke, bleeding, or cardiogenic shock. For secondary outcomes in SURTAVI, the treatment effect was similar for all outcomes [i.e., death, stroke, aortic valve replacement, myocardial infarction, and major adverse cardiovascular events (MACE)]. Together, these results suggested the possible utility of claims to assess mortality and procedural outcomes in trial follow-up but suggest caution with use of claims to ascertain some non-procedural endpoints.

#### Case study 4: linkage of RCT data to medical claims to evaluate transportability of treatment effect to real world populations in a US setting

In the same set of linked trials and Medicare claims as in case study three above, as part of the EXTEND study, investigators assessed how trial results might change if participants reflected the broader (“real-world”) Medicare population. Demographics, comorbidities, and frailty indices were compared between trial and real-world patients; trial participation was modeled via logistic regression to generate propensity scores; and inverse probability weighting was applied to reweight characteristics of trial participants to resemble those of the general Medicare population. Subsequently, treatment effect estimates were evaluated in the reweighted trial data and compared to those from the original trial. In the CoreValve pivotal trials, trial patients were overall similar to those in Medicare but were more likely to have hypertension and coagulopathy and Medicare patients were more likely to have congestive heart failure and frailty. The estimated real-world treatment effect of TAVR was an 11.4% reduction in death or stroke, exceeding the observed trial reduction of 8.4%. By contrast, in the DAPT study where trial data was reweighted using data from the linked National Cardiovascular Data Registry (NCDR) CathPCI registry, there was attenuation and loss of a significant effect of the intervention (e.g., prolonged DAPT) on reducing stent thrombosis, MACE but a similar increase in bleeding risk. These findings highlight healthy volunteer bias and indicate that transportability of trial effects to routine practice varies by intervention and outcome.

### Actionable recommendations

The four case studies in this commentary, spanning different indications, types of interventions, and regional settings, together with the broader literature, support a set of actionable recommendations for RCT–RWD linkage studies for product development, organized around the four interdependent domains: decision context; linkage performance and properties; governance and privacy-preserving implementation; and operational feasibility and availability of reliable and relevant data sources.

#### Align RCT-RWD linkage to the decision context

Sponsors should prospectively specify the regulatory, HTA, payer, or clinical decision the linked evidence is intended to inform, as well as the specific research question it is intended to answer–such as treatment-effect durability, transportability, safety surveillance, or cost-effectiveness. Case studies 3 and 4 (EXTEND/ CoreValve) illustrate the distinction: the same linked dataset supported reproduction of the trial’s treatment-effect estimate within the trial population and transportability to the broader Medicare population, each requiring distinct analytic frameworks and identifiability assumptions (Berger et al., 2017; Degtiar and Rose, 2023).

#### Plan, measure, and report RCT-RWD linkage performance

RCT-RWD linkage quality should be assessed quantitatively and reported transparently, consistent with the ISPE linkage reporting framework (Pratt et al., 2020; Rivera et al., 2020) and CONSORT-ROUTINE (Kwakkenbos et al., 2021). Studies should pre-specify overall and subgroup-stratified match rates, linkage performance metrics where a reference standard is available, and the false-match/ missed-match trade-off. For example, case study three showed that Medicare claims reproduced the trial treatment-effect estimate for mortality and procedural endpoints but diverged for stroke, bleeding, and cardiogenic shock. Because linkage failure is rarely random, comparison of linked and non-linked subpopulations should be standard (Harron et al., 2017), and the proportion of consented participants ultimately linked to RWD should itself be a pre-specified operational metric, with explicit analytic approaches for scenarios in which the achieved linkage rate falls materially short of expectations. For example, case study 2 (ConquerRSV/ ROSE) showed only 38.6% of the 67% who consented were ultimately linked to claims, largely due to payer fragmentation. No current regulatory guidance or consensus standard establishes a minimum acceptable linkage rate. Instead, acceptability should be evaluated in a decision-specific manner, taking into account the intended clinical and regulatory use and whether linkage failure is differential by patient or other clinically important characteristics. Reassessing balance between treatment groups in the final linked study population is also prudent as imbalances suggest that the trial randomization is no longer sufficient in controlling potential confounders. In case study 2 (ConquerRSV/ ROSE), the RSV-vaccinated and placebo groups following linkage remained balanced on most characteristics assessed despite attrition.

#### Document governance and data privacy-preserving implementation to support inspection readiness

Although existing guidance on RWD provenance, source data integrity, and good clinical practice (EMA, 2025; FDA, 2023; ICH, 2025) establishes relevant principles, none addresses RCT–RWD linkage governance specifically. Inspection readiness therefore depends on traceable governance documentation that operationalizes these principles for the linkage setting: a data-flow and provenance diagram, a Data Protection Impact Assessment or equivalent under the governing framework (GDPR, HIPAA, APPI), a linkage identifier management plan, and a pre-specified authoritative source for each variable and time period with reconciliation rules. Country-level differences in governance, consent, and identifier availability must be planned for even within a single trial: case study 1 (FUTURE II LTFU) achieved near-complete linkage via universal Personal Identity Numbers (Enerly et al., 2020), an infrastructure that does not port to fragmented systems such as in the U.S. RCT-RWD linkage studies should prioritize patient-centered design, including consent language developed with patient and stakeholder input, explicit scope for secondary use, clear procedures for withdrawing consent, including after linkage has occurred, and participant transparency (Arellano et al., 2023; Eckrote et al., 2024).

#### Design for operational feasibility and reuse across RCT-RWD linkage studies

Integrating RCT-RWD linkage planning into the trial protocol from the outset–including consent, identifier collection, site training, and infrastructure selection–is the most consequential operational recommendation sponsors can implement directly. Sponsors expecting to conduct multiple linkage studies over time should invest in reusable infrastructure that can support multiple studies and be shared across teams, such as a tokenization framework, pre-negotiated data use agreements, validated trusted research environments, and consistent metadata standards, rather than rebuilding these for each study. Case studies 3 and 4 (EXTEND) illustrate this principle: the same linked RCT trial–Medicare claims platform supported both reproduction of randomized treatment effects in the trial population and transportability analyses to the broader Medicare population (Kiernan et al., 2022; Marsolo et al., 2023). Long-term follow up studies must also consider and plan for changes over time (e.g., evolving standards of care, shifts in available comparator treatments, and transitions between coding systems (ICD-9 to 10–11) by specifying in advance, at least at a high level, how these issues would be addressed in the analysis.

### Future development/conclusion

RCT–RWD linkage offers efficient opportunities to extend the evidence generated by conventional RCTs, enabling assessment of real-world effectiveness, safety, long-term outcomes, outcomes not generally assessed in trials (e.g., economic outcomes, healthcare resource utilization), and generalizability. As the case studies presented here illustrate, successful linkage depends on alignment across the four interdependent domains introduced early in development: a clearly defined decision context and question to be answered; quantitative assessment and transparent reporting of linkage performance; traceable governance documentation; and operational planning integrated into trial design rather than added after the trial is already underway. Applications of artificial intelligence and machine learning to linkage (Janoudi et al., 2024) should be pursued with caution and with explicit attention to the well-documented risk that linkage failure is not random and can fall disproportionately on groups under-represented in real-world data (Grath-Lone et al., 2021).

Progress beyond the current state will require global health authority guidance specific to linking clinical trials with routinely collected data, shared benchmarks for evaluating linkage approaches, routine reporting and assessments of lessons learned and impact of clinical trial use cases with linkages to routine healthcare data sources on regulatory and reimbursement decisions. Linkage of clinical trial data with routinely collected data sources can meaningfully accelerate clinical development and inform health authority and other clinical decision making.
