# Supplementary material for: Linking clinical trial data with real-world data for enhanced clinical evidence generation: methodological considerations and recommendations
Source: Front Pharmacol. 2026 Aug 7;17:1887249. doi: 10.3389/fphar.2026.1887249 (PMC13494585; doi:10.3389/fphar.2026.1887249)
Supplement: Supplementary file 1 [file Table1.docx]

**Supplemental Table 1.** Baseline demographic and clinical characteristics among ConquerRSV participants who had consented to ROSE and been successfully linked with claims using tokenization.

|  | **Investigational vaccine** | **Placebo** | **ASD** |
| --- | --- | --- | --- |
|  | **N = 2,115** | **N = 2,127** |  |
| **Days covered by healthcare insurance prior to injection date** | | | |
| Mean (SD) | 905.8 (559.25) | 882.4 (542.30) | 0.042 |
| Median (Q1, Q3) | 873 (669, 973) | 862 (604, 964) |  |
| **Age** |  |  |  |
| Mean (SD) | 69.9 (6.16) | 69.8 (6.18) | 0.013 |
| Median (Q1, Q3) | 69 (65, 74) | 69 (66, 73) |  |
| **Sex, n (%)** | | | |
| Female | 1008 (47.7) | 962 (45.2) | 0.058 |
| Male | 1018 (48.1) | 1091 (51.3) | 0.072 |
| Unknown/missing | 89 (4.2) | 74 (3.5) | 0.056 |
| **Race/ethnicity, n (%)** | | | |
| White | 1339 (63.3) | 1374 (64.6) | 0.028 |
| Black | 266 (12.6) | 284 (13.4) | 0.031 |
| Hispanic/Latino | 52 (2.5) | 36 (1.7) | 0.083 |
| Asian | 34 (1.6) | 33 (1.6) | 0.006 |
| Other Race | 40 (1.9) | 39 (1.8) | 0.006 |
| Unknown/missing | 384 (18.2) | 361 (17.0) | 0.042 |
| **Geographic region, n (%)** | | | |
| South | 850 (40.2) | 851 (40.0) | 0.004 |
| Midwest | 345 (16.3) | 338 (15.9) | 0.016 |
| West | 639 (30.2) | 685 (32.2) | 0.054 |
| Northeast | 185 (8.7) | 172 (8.1) | 0.033 |
| Unknown/Missing | 96 (4.5) | 81 (3.8) | 0.053 |
| **Payer, n (%)** | | | |
| Commercial | 204 (9.6) | 199 (9.4) | 0.014 |
| Managed Medicaid | 194 (9.2) | 185 (8.7) | 0.023 |
| Medicare Advantage | 311 (14.7) | 284 (13.4) | 0.054 |
| Medicare FFS | 1406 (66.5) | 1459 (68.6) | 0.045 |
| **Injection calendar year - month, n (%)** | | | |
| 2021 | 104 (4.9) | 82 (3.9) | 0.077 |
| November | 17 (0.8) | 13 (0.6) | 0.035 |
| December | 87 (4.1) | 69 (3.2) | 0.069 |
| 2022 | 2011 (95.1) | 2045 (96.1) | 0.021 |
| January | 111 (5.2) | 99 (4.7) | 0.039 |
| February | 102 (4.8) | 92 (4.3) | 0.034 |
| March | 155 (7.3) | 167 (7.9) | 0.027 |
| April | 140 (6.6) | 160 (7.5) | 0.047 |
| May | 201 (9.5) | 224 (10.5) | 0.046 |
| June | 236 (11.2) | 218 (10.2) | 0.041 |
| July | 366 (17.3) | 357 (16.8) | 0.019 |
| August | 346 (16.4) | 378 (17.8) | 0.050 |
| September | 218 (10.3) | 229 (10.8) | 0.020 |
| October | 125 (5.9) | 106 (5.0) | 0.059 |
| November/December | 11 (0.5) | 15 (0.7) | 0.031 |
| **Comorbid conditions, n (%)** | | | |
| Advanced Liver Disease | 19 (0.9) | 20 (0.9) | 0.006 |
| Advanced Renal Disease | 218 (10.3) | 225 (10.6) | 0.012 |
| Asthma | 175 (8.3) | 159 (7.5) | 0.042 |
| CHF | 112 (5.3) | 124 (5.8) | 0.032 |
| COPD | 215 (10.2) | 211 (9.9) | 0.011 |
| Diabetes | 463 (21.9) | 386 (18.1) | 0.130 |
| **Claims based frailty index** | | | |
| Mean (SD) | 0.5 (1.06) | 0.5 (1.08) | 0.019 |
| Median (Q1, Q3) | 0.2 (0.1, 0.5) | 0.2 (0.1, 0.5) |  |
| Categories, n (%) |  |  |  |
| Non-frail | 975 (46.1) | 1016 (47.8) | 0.039 |
| Prefrail | 236 (11.2) | 250 (11.8) | 0.025 |
| Frail | 904 (42.7) | 861 (40.5) | 0.056 |

Abbreviation: ASD = absolute standardized mean difference; CHF = congestive heart failure; COPD = chronic obstructive pulmonary disease; SD = standard deviation.
